# *catena*-Poly[[triphenyl­tin(IV)]-μ-phenyl­phosphinato-κ^2^ *O*:*O*′]

**DOI:** 10.1107/S1600536811043625

**Published:** 2011-11-05

**Authors:** Tidiane Diop, Libasse Diop, Gabriele Kociok-Köhn, Kieran C. Molloy, Helen Stoeckli-Evans

**Affiliations:** a Laboratoire de Chimie Minérale et Analytique, Département de Chimie, Faculté des Sciences et Techniques–Université Cheikh Anta Diop, Dakar, Senegal; b Department of Chemistry, University of Bath, Claverton Down, Bath BA2 7AY, England; c Institute of Physics, University of Neuchâtel, Rue Emile-Argand 11, CH-2000 Neuchâtel, Switzerland

## Abstract

In the structure of the title coordination polymer, [Sn(C_6_H_5_)_3_(C_6_H_6_O_2_P)]_*n*_ or [PhP(H)O_2_Sn^IV^(Ph)_3_]_*n*_, the Sn^IV^ atom is five-coordinate, with the SnC_3_O_2_ framework in a *trans* trigonal–bipyramidal arrangement having the PhP(H)O_2_^−^ anions in apical positions. In the crystal, neighbouring polymer chains are linked *via* C—H⋯π inter­actions, forming a two-dimensional network lying parallel to (001).

## Related literature

For medical applications of tin(IV) compounds, see: Evans & Karpel (1985 ▶); Kapoor *et al.* (2005 ▶); Yin & Wang (2004 ▶). For literature on new organotin compounds, see: Chandrasekhar *et al.* (2003 ▶); Davies & Smith (1982 ▶); Zhang *et al.* (2006 ▶). For work in this field carried out by the authors, see: Diassé-Sarr *et al.* (1997 ▶); Diop *et al.* (2002 ▶, 2003 ▶); Diallo *et al.* (2009 ▶). For related structures, see: Molloy *et al.* (1981 ▶); Adair *et al.* (2003 ▶).
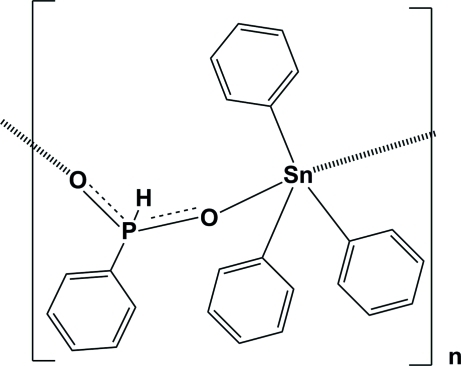


## Experimental

### 

#### Crystal data

[Sn(C_6_H_5_)_3_(C_6_H_6_O_2_P)]
*M* *_r_* = 491.07
Orthorhombic, 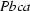

*a* = 14.0108 (6) Å
*b* = 11.7674 (7) Å
*c* = 25.7068 (12) Å
*V* = 4238.3 (4) Å^3^
*Z* = 8
Mo *K*α radiation
μ = 1.30 mm^−1^
*T* = 173 K
0.18 × 0.13 × 0.10 mm

#### Data collection

Stoe IPDS II diffractometer
Absorption correction: multi-scan (*MULscanABS* in *PLATON*; Spek, 2009 ▶) *T*_min_ = 0.973, *T*_max_ = 1.000
27270 measured reflections
3829 independent reflections
2467 reflections with *I* > 2σ(*I*)
*R*_int_ = 0.117

#### Refinement

*R*[*F* ^2^ > 2σ(*F* ^2^)] = 0.052
*wR*(*F* ^2^) = 0.079
*S* = 1.00
3829 reflections
257 parameters
H atoms treated by a mixture of independent and constrained refinement
Δρ_max_ = 0.47 e Å^−3^
Δρ_min_ = −0.68 e Å^−3^

### 

Data collection: *X-AREA* (Stoe & Cie, 2009 ▶); cell refinement: *X-AREA*; data reduction: *X-RED32* (Stoe & Cie, 2009 ▶); program(s) used to solve structure: *SHELXS97* (Sheldrick, 2008 ▶); program(s) used to refine structure: *SHELXL97* (Sheldrick, 2008 ▶); molecular graphics: *PLATON* (Spek, 2009 ▶) and *Mercury* (Macrae *et al.*, 2008 ▶); software used to prepare material for publication: *SHELXL97*, *PLATON* and *publCIF* (Westrip, 2010 ▶).

## Supplementary Material

Crystal structure: contains datablock(s) I, global. DOI: 10.1107/S1600536811043625/mw2028sup1.cif

Structure factors: contains datablock(s) I. DOI: 10.1107/S1600536811043625/mw2028Isup2.hkl

Additional supplementary materials:  crystallographic information; 3D view; checkCIF report

## Figures and Tables

**Table 1:** Hydrogen-bond geometry (Å, °) *Cg*1 is the centroid of the C19–C24 ring.

*D*—H⋯*A*	*D*—H	H⋯*A*	*D*⋯*A*	*D*—H⋯*A*
C9—H9⋯*Cg*1^i^	0.95	2.79	3.656 (9)	151
C18—H18⋯*Cg*1^ii^	0.95	2.91	3.714 (6)	143

Symmetry codes: (i) 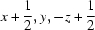; (ii) 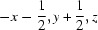.

**Fig. 1.**
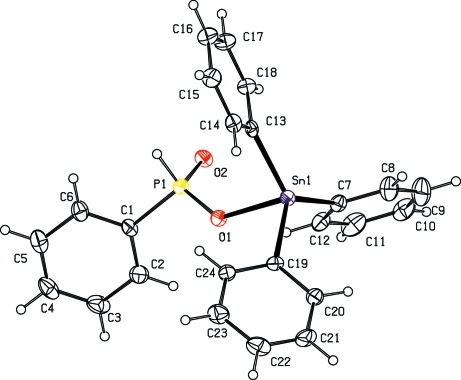
A view of the asymmetric unit of the title compound, showing the numbering scheme and displacement ellipsoids drawn at the 50% probability level.

**Fig. 2.**
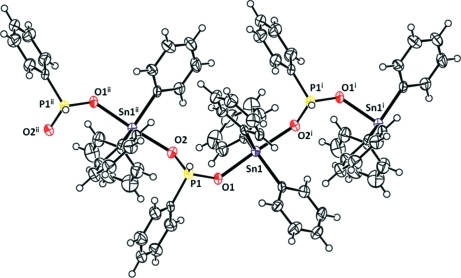
A view normal to (001) of the polymer chain of the title compound [Symmetry codes: (i) -x - 1/2, y - 1/2, z; (ii) -x - 1/2, y + 1/2, z; displacement ellipsoids are drawn at the 50% probability level].

**Fig. 3.**
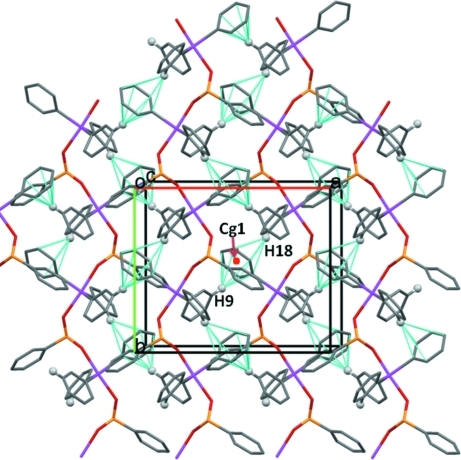
A view along the c axis of the crystal packing of the title compound showing the weak C—H···π interactions [represented by the H···C dashed cyan lines; Sn violet; P yellow; O red; H grey ball; Cg1 = centroid of ring (C19—C24)].

## supplementary crystallographic information

### Comment

As some compounds belonging to the organotin (IV) family have been found to be the subject of applications in medicine, agriculture, industry (Evans & Karpel, 1985; Kapoor *et al.*, 2005; Yin & Wang, 2004), many groups have been involved in the search for new organotin compounds (Davies & Smith, 1982; Zhang *et al.*, 2006; Chandrasekhar *et al.*, 2003). Our group has published a number of papers in this field (Diassé-Sarr *et al.*, 1997; Diop *et al.*, 2003; Diop *et al.*, 2002; Diallo *et al.*, 2009). In a continuation of this work we initiated the study of the interaction between Cy_2_NH_2_PhP(H)O_2_ and Sn(Ph)_3_Cl, which has led to the synthesis of the title coordination polymer.

The structure of the asymmetric unit of the title compound is illustrated in Fig. 1. The molecular units associate to form an infinite one-dimensional polymer (Fig. 2) in which trimethyltin(IV) groups are axially bridged by –O—P—O– linkages of the phenylphosphinate ligand to yield an almost perfect trigonal bipyramid at the tin(IV) atom; with equatorial location of the phenyl groups and axial disposition of the oxygenated ligand.

The sum of the angles at atom Sn1 by the *ipso*-carbons [124.1 (2), 119.4 (3), 116.4 (3) °] is 359.9°. The corresponding axial O1—Sn—O2 angle is 175.99 (15) °, indicating a slight deviation from linearity. The two axial Sn—O distances, [Sn1—O1 2.241 (4) Å and Sn—O2 2.237 (3) Å], are longer than the Sn—O axial distances [2.116 (2) Å and 2.132 (3) Å] observed in *catena*-(µ_2_-phenylphosphinato-O,*O*')-choro-tin(II) [Adair *et al.*, 2003]. The two P—O distances of the bridging O1—P1—O2 moieties are also almost equal [P1—O1 1.514 (4) Å and P1—O2 1.501 (4) Å]. The geometry around the phosphorus atom is a distorted tetrahedron with bond angles ranging from 114.4 (2)° for O1—P1—O2 to 103 (2)° for C1—P1—H1. The P1—H1 distance is 1.33 (5) Å, similar to the same distance, of 1.39 (7) Å, observed in the compound mentioned above.

In the crystal, neighbouring chains are linked *via* C—H···π interactions, involving the phenyl ring (C19—C24). This results in the formation of a two-dimensional network structure lying parallel to the *ab*-plane (Table 1 and Fig. 3).

Footnote to Table 1: *Cg*1 is the centroid of ring (C19—C24).

### Experimental

Synthesis: Cy_2_NH_2_Ph(H)PO_2_ (*L*) was obtained on neutralizing phenylphosphinic acid with dicyclohexylammine, in 1:2 ratio, in water; a white powder was collected after evaporation at 333 K. When (*L*) was mixed with Sn(Ph)_3_Cl (1:1 ratio, *M*.p. +533 K), both in ethanol, a white precipitate formed and the solution was stirred for a further 2 h. The mixture was then filtered and the solid dissolved in 25 ml of slightly hydrated methanol. The solution was then left for the solvent to slowly evaporate giving colourless crystals, suitable for X-ray diffraction analysis, of the title compound. Reaction: Cy_2_NH_2_Ph(H)PO_2_ + Sn(Ph)_3_Cl --> PhP(H)O_2_Sn(Ph)_3_ + Cy_2_NH_2_Cl. The same compound could be obtained by refluxing trimethyltin chloride with phenylphosphinic acid in water: Ph(H)PO_2_H + Sn(Ph)_3_Cl --> PhP(H)O_2_Sn(Ph)_3_ + HCl.

### Refinement

The PH H-atom was located in a difference Fourier map and was refined with *U*_iso_(H) = 1.2*U*_eq_(P); [P—H = 1.33 (5) Å]. The C-bound H atoms were included in calculated positions and treated as riding atoms: C—H = 0.95 Å with *U*_iso_(H) = 1.2*U*_eq_(C).

### Crystal data

**Table:**

[Sn(C_6_H_5_)_3_(C_6_H_6_O_2_P)]	*F*(000) = 1968
*M**_r_* = 491.07	*D*_x_ = 1.539 Mg m^−^^3^
Orthorhombic, *P**b**c**a*	Mo *K*α radiation, λ = 0.71073 Å
Hall symbol: -P 2ac 2ab	Cell parameters from 10452 reflections
*a* = 14.0108 (6) Å	θ = 1.6–26.1°
*b* = 11.7674 (7) Å	µ = 1.30 mm^−^^1^
*c* = 25.7068 (12) Å	*T* = 173 K
*V* = 4238.3 (4) Å^3^	Rod, colourless
*Z* = 8	0.18 × 0.13 × 0.10 mm

### Data collection

**Table:**

Stoe IPDS II diffractometer	3829 independent reflections
Radiation source: fine-focus sealed tube	2467 reflections with *I* > 2σ(*I*)
plane graphite	*R*_int_ = 0.117
φ and ω scans	θ_max_ = 25.3°, θ_min_ = 1.6°
Absorption correction: multi-scan (MULscanABS in *PLATON*; Spek, 2009)	*h* = −16→16
*T*_min_ = 0.973, *T*_max_ = 1.000	*k* = −14→14
27270 measured reflections	*l* = −28→30

### Refinement

**Table:**

Refinement on *F*^2^	Secondary atom site location: difference Fourier map
Least-squares matrix: full	Hydrogen site location: inferred from neighbouring sites
*R*[*F*^2^ > 2σ(*F*^2^)] = 0.052	H atoms treated by a mixture of independent and constrained refinement
*wR*(*F*^2^) = 0.079	*w* = 1/[σ^2^(*F*_o_^2^) + (0.0275*P*)^2^] where *P* = (*F*_o_^2^ + 2*F*_c_^2^)/3
*S* = 1.00	(Δ/σ)_max_ = 0.001
3829 reflections	Δρ_max_ = 0.47 e Å^−^^3^
257 parameters	Δρ_min_ = −0.68 e Å^−^^3^
0 restraints	Extinction correction: *SHELXL97* (Sheldrick, 2008), Fc^*^=kFc[1+0.001xFc^2^λ^3^/sin(2θ)]^-1/4^
Primary atom site location: structure-invariant direct methods	Extinction coefficient: 0.00036 (5)

### Special details

**Table:**

Geometry. Bond distances, angles *etc*. have been calculated using the rounded fractional coordinates. All su's are estimated from the variances of the (full) variance-covariance matrix. The cell e.s.d.'s are taken into account in the estimation of distances, angles and torsion angles
Refinement. The PH H-atom was located in a difference Fourier map and was refined with *U*_iso_(H) = 1.2U_e_q(P). The C-bound H-atoms were included in calculated positions and treated as riding atoms: C—H = 0.95 Å for CH(aromatic), with *U*_iso_(H) = 1.2U_e_q(C).

### Fractional atomic coordinates and isotropic or equivalent isotropic displacement parameters (Å^2^)

**Table:**

	*x*	*y*	*z*	*U*_iso_*/*U*_eq_	
Sn1	−0.29327 (3)	−0.35558 (3)	0.36284 (1)	0.0260 (1)	
P1	−0.36921 (12)	−0.08232 (12)	0.38935 (6)	0.0286 (5)	
O1	−0.3855 (3)	−0.2003 (3)	0.36764 (17)	0.0295 (11)	
O2	−0.2923 (3)	−0.0161 (2)	0.36231 (17)	0.0350 (11)	
C1	−0.4806 (4)	−0.0060 (4)	0.3878 (3)	0.029 (2)	
C2	−0.5372 (5)	−0.0034 (5)	0.3438 (3)	0.047 (3)	
C3	−0.6217 (5)	0.0586 (6)	0.3440 (3)	0.053 (3)	
C4	−0.6498 (5)	0.1153 (5)	0.3884 (3)	0.052 (3)	
C5	−0.5930 (5)	0.1151 (5)	0.4317 (3)	0.050 (3)	
C6	−0.5077 (5)	0.0548 (5)	0.4313 (3)	0.040 (2)	
C7	−0.2349 (4)	−0.3088 (5)	0.2893 (2)	0.0317 (19)	
C8	−0.1608 (5)	−0.3689 (6)	0.2669 (3)	0.050 (3)	
C9	−0.1227 (6)	−0.3364 (7)	0.2197 (3)	0.065 (3)	
C10	−0.1576 (6)	−0.2451 (6)	0.1937 (3)	0.054 (3)	
C11	−0.2315 (6)	−0.1838 (6)	0.2150 (3)	0.059 (3)	
C12	−0.2705 (5)	−0.2148 (5)	0.2623 (2)	0.041 (2)	
C13	−0.2300 (4)	−0.3139 (4)	0.4356 (2)	0.0260 (18)	
C14	−0.2518 (5)	−0.3764 (4)	0.4803 (2)	0.0323 (17)	
C15	−0.2086 (5)	−0.3528 (5)	0.5273 (2)	0.0400 (17)	
C16	−0.1414 (5)	−0.2654 (5)	0.5310 (3)	0.042 (3)	
C17	−0.1206 (5)	−0.2024 (5)	0.4872 (2)	0.039 (2)	
C18	−0.1629 (4)	−0.2252 (5)	0.4404 (2)	0.0327 (19)	
C19	−0.4177 (4)	−0.4597 (4)	0.3655 (3)	0.0273 (16)	
C20	−0.4303 (5)	−0.5480 (5)	0.3302 (3)	0.038 (2)	
C21	−0.5101 (5)	−0.6171 (5)	0.3344 (3)	0.047 (3)	
C22	−0.5766 (5)	−0.5986 (5)	0.3719 (3)	0.050 (3)	
C23	−0.5660 (5)	−0.5107 (6)	0.4068 (3)	0.048 (3)	
C24	−0.4866 (4)	−0.4413 (5)	0.4031 (2)	0.034 (2)	
H1	−0.351 (4)	−0.089 (4)	0.440 (2)	0.0340*	
H2	−0.51850	−0.04380	0.31350	0.0570*	
H3	−0.66010	0.06180	0.31360	0.0640*	
H4	−0.70900	0.15470	0.38890	0.0620*	
H5	−0.61170	0.15600	0.46190	0.0590*	
H6	−0.46790	0.05530	0.46120	0.0480*	
H8	−0.13570	−0.43360	0.28430	0.0600*	
H9	−0.07140	−0.37860	0.20510	0.0770*	
H10	−0.13120	−0.22350	0.16120	0.0650*	
H11	−0.25600	−0.11970	0.19700	0.0710*	
H12	−0.32180	−0.17200	0.27650	0.0500*	
H14	−0.29720	−0.43620	0.47820	0.0390*	
H15	−0.22460	−0.39600	0.55730	0.0480*	
H16	−0.11060	−0.24970	0.56310	0.0510*	
H17	−0.07590	−0.14200	0.48960	0.0460*	
H18	−0.14700	−0.18070	0.41070	0.0390*	
H20	−0.38480	−0.56080	0.30350	0.0450*	
H21	−0.51820	−0.67840	0.31080	0.0570*	
H22	−0.63100	−0.64670	0.37400	0.0600*	
H23	−0.61240	−0.49800	0.43310	0.0570*	
H24	−0.47930	−0.37990	0.42670	0.0410*	

### Atomic displacement parameters (Å^2^)

**Table:**

	*U*^11^	*U*^22^	*U*^33^	*U*^12^	*U*^13^	*U*^23^
Sn1	0.0255 (2)	0.0192 (2)	0.0334 (2)	−0.0004 (2)	0.0006 (2)	0.0008 (2)
P1	0.0288 (9)	0.0191 (7)	0.0380 (9)	−0.0004 (7)	−0.0023 (8)	−0.0018 (7)
O1	0.031 (2)	0.0156 (16)	0.042 (2)	−0.0002 (15)	−0.002 (2)	−0.0021 (18)
O2	0.029 (2)	0.0209 (16)	0.055 (2)	−0.0052 (18)	0.002 (3)	−0.0016 (19)
C1	0.027 (4)	0.014 (3)	0.047 (4)	0.002 (3)	0.002 (3)	0.003 (3)
C2	0.041 (5)	0.037 (3)	0.064 (5)	0.006 (3)	−0.010 (4)	−0.012 (3)
C3	0.042 (5)	0.041 (4)	0.076 (5)	0.008 (3)	−0.018 (4)	0.003 (4)
C4	0.033 (4)	0.026 (4)	0.096 (6)	0.004 (3)	0.007 (4)	0.003 (3)
C5	0.044 (5)	0.041 (4)	0.064 (5)	0.006 (3)	0.015 (4)	−0.001 (3)
C6	0.041 (4)	0.031 (3)	0.048 (4)	0.006 (3)	0.006 (3)	0.002 (3)
C7	0.036 (4)	0.025 (3)	0.034 (3)	−0.003 (3)	0.001 (3)	−0.001 (3)
C8	0.061 (5)	0.041 (4)	0.048 (4)	0.005 (4)	0.013 (4)	0.003 (3)
C9	0.076 (6)	0.065 (5)	0.053 (5)	0.001 (5)	0.030 (4)	0.001 (4)
C10	0.070 (6)	0.063 (5)	0.030 (4)	−0.022 (4)	0.010 (4)	0.000 (4)
C11	0.086 (7)	0.052 (4)	0.040 (4)	−0.009 (4)	−0.005 (4)	0.007 (3)
C12	0.053 (5)	0.036 (3)	0.035 (4)	0.003 (3)	−0.006 (3)	−0.006 (3)
C13	0.027 (4)	0.017 (2)	0.034 (3)	0.005 (2)	0.004 (3)	−0.001 (2)
C14	0.035 (3)	0.019 (3)	0.043 (3)	−0.001 (2)	0.006 (3)	0.003 (2)
C15	0.043 (3)	0.045 (3)	0.032 (3)	−0.001 (4)	0.002 (3)	0.007 (3)
C16	0.045 (5)	0.045 (4)	0.037 (4)	−0.004 (3)	−0.006 (3)	0.000 (3)
C17	0.044 (4)	0.031 (3)	0.041 (4)	−0.010 (3)	−0.008 (3)	0.004 (3)
C18	0.036 (4)	0.031 (3)	0.031 (3)	−0.003 (3)	−0.004 (3)	0.005 (3)
C19	0.030 (3)	0.015 (2)	0.037 (3)	0.001 (2)	−0.010 (4)	0.001 (3)
C20	0.035 (4)	0.030 (3)	0.048 (4)	0.000 (3)	−0.003 (3)	−0.008 (3)
C21	0.051 (5)	0.026 (4)	0.064 (5)	−0.007 (3)	−0.012 (4)	−0.012 (3)
C22	0.041 (4)	0.034 (3)	0.075 (6)	−0.014 (3)	−0.005 (4)	0.009 (4)
C23	0.040 (5)	0.045 (4)	0.059 (5)	−0.013 (3)	0.005 (4)	0.002 (4)
C24	0.031 (4)	0.030 (3)	0.042 (4)	−0.006 (3)	0.002 (3)	−0.006 (3)

### Geometric parameters (Å, °)

**Table:**

Sn1—O1	2.241 (4)	C19—C24	1.383 (9)
Sn1—C7	2.132 (5)	C19—C20	1.391 (9)
Sn1—C13	2.127 (5)	C20—C21	1.387 (9)
Sn1—C19	2.132 (5)	C21—C22	1.358 (10)
Sn1—O2^i^	2.237 (3)	C22—C23	1.377 (10)
P1—O1	1.514 (4)	C23—C24	1.383 (9)
P1—O2	1.501 (4)	C2—H2	0.9500
P1—C1	1.801 (6)	C3—H3	0.9500
P1—H1	1.33 (5)	C4—H4	0.9500
C1—C2	1.382 (10)	C5—H5	0.9500
C1—C6	1.381 (10)	C6—H6	0.9500
C2—C3	1.391 (10)	C8—H8	0.9500
C3—C4	1.380 (10)	C9—H9	0.9500
C4—C5	1.368 (11)	C10—H10	0.9500
C5—C6	1.390 (10)	C11—H11	0.9500
C7—C12	1.398 (8)	C12—H12	0.9500
C7—C8	1.382 (9)	C14—H14	0.9500
C8—C9	1.380 (11)	C15—H15	0.9500
C9—C10	1.357 (11)	C16—H16	0.9500
C10—C11	1.376 (11)	C17—H17	0.9500
C11—C12	1.382 (10)	C18—H18	0.9500
C13—C14	1.398 (7)	C20—H20	0.9500
C13—C18	1.410 (8)	C21—H21	0.9500
C14—C15	1.380 (8)	C22—H22	0.9500
C15—C16	1.398 (9)	C23—H23	0.9500
C16—C17	1.379 (9)	C24—H24	0.9500
C17—C18	1.368 (8)		
			
O1—Sn1—C7	93.41 (19)	C19—C20—C21	119.3 (7)
O1—Sn1—C13	90.22 (17)	C20—C21—C22	121.0 (6)
O1—Sn1—C19	89.73 (17)	C21—C22—C23	120.6 (6)
O1—Sn1—O2^i^	175.99 (15)	C22—C23—C24	119.0 (6)
C7—Sn1—C13	124.1 (2)	C19—C24—C23	121.1 (6)
C7—Sn1—C19	119.4 (3)	C1—C2—H2	120.00
O2^i^—Sn1—C7	90.41 (19)	C3—C2—H2	120.00
C13—Sn1—C19	116.4 (3)	C2—C3—H3	120.00
O2^i^—Sn1—C13	88.66 (17)	C4—C3—H3	120.00
O2^i^—Sn1—C19	87.32 (17)	C3—C4—H4	120.00
O1—P1—O2	114.4 (2)	C5—C4—H4	120.00
O1—P1—C1	108.6 (3)	C4—C5—H5	120.00
O2—P1—C1	110.7 (2)	C6—C5—H5	120.00
O1—P1—H1	110 (2)	C1—C6—H6	120.00
O2—P1—H1	110 (2)	C5—C6—H6	120.00
C1—P1—H1	103 (2)	C7—C8—H8	119.00
Sn1—O1—P1	132.9 (3)	C9—C8—H8	120.00
Sn1^ii^—O2—P1	145.1 (2)	C8—C9—H9	120.00
P1—C1—C2	121.8 (5)	C10—C9—H9	119.00
C2—C1—C6	119.6 (6)	C9—C10—H10	120.00
P1—C1—C6	118.6 (5)	C11—C10—H10	120.00
C1—C2—C3	119.8 (7)	C10—C11—H11	120.00
C2—C3—C4	120.0 (7)	C12—C11—H11	120.00
C3—C4—C5	120.4 (6)	C7—C12—H12	120.00
C4—C5—C6	119.7 (7)	C11—C12—H12	120.00
C1—C6—C5	120.5 (7)	C13—C14—H14	119.00
Sn1—C7—C8	121.7 (5)	C15—C14—H14	120.00
Sn1—C7—C12	120.5 (4)	C14—C15—H15	120.00
C8—C7—C12	117.8 (5)	C16—C15—H15	120.00
C7—C8—C9	121.0 (7)	C15—C16—H16	121.00
C8—C9—C10	120.9 (7)	C17—C16—H16	121.00
C9—C10—C11	119.4 (7)	C16—C17—H17	119.00
C10—C11—C12	120.6 (7)	C18—C17—H17	119.00
C7—C12—C11	120.3 (6)	C13—C18—H18	120.00
Sn1—C13—C14	120.7 (4)	C17—C18—H18	120.00
C14—C13—C18	117.6 (5)	C19—C20—H20	120.00
Sn1—C13—C18	121.7 (4)	C21—C20—H20	120.00
C13—C14—C15	121.2 (5)	C20—C21—H21	119.00
C14—C15—C16	120.2 (6)	C22—C21—H21	120.00
C15—C16—C17	118.8 (6)	C21—C22—H22	120.00
C16—C17—C18	121.4 (6)	C23—C22—H22	120.00
C13—C18—C17	120.7 (5)	C22—C23—H23	121.00
Sn1—C19—C20	120.8 (5)	C24—C23—H23	120.00
Sn1—C19—C24	120.2 (4)	C19—C24—H24	119.00
C20—C19—C24	119.0 (5)	C23—C24—H24	119.00
			
C7—Sn1—O1—P1	−89.4 (4)	O1—P1—C1—C6	134.2 (5)
C13—Sn1—O1—P1	34.8 (4)	O2—P1—C1—C2	78.1 (5)
C19—Sn1—O1—P1	151.2 (4)	O2—P1—C1—C6	−99.4 (5)
O1—Sn1—C7—C8	172.5 (5)	P1—C1—C2—C3	−178.7 (5)
O1—Sn1—C7—C12	−7.0 (5)	C6—C1—C2—C3	−1.2 (9)
C13—Sn1—C7—C8	79.9 (6)	P1—C1—C6—C5	179.9 (5)
C13—Sn1—C7—C12	−99.6 (5)	C2—C1—C6—C5	2.3 (9)
C19—Sn1—C7—C8	−95.9 (5)	C1—C2—C3—C4	−1.4 (10)
C19—Sn1—C7—C12	84.6 (5)	C2—C3—C4—C5	2.8 (10)
O2^i^—Sn1—C7—C8	−8.7 (5)	C3—C4—C5—C6	−1.7 (10)
O2^i^—Sn1—C7—C12	171.8 (5)	C4—C5—C6—C1	−0.9 (10)
O1—Sn1—C13—C14	107.3 (5)	Sn1—C7—C8—C9	−178.9 (6)
O1—Sn1—C13—C18	−74.6 (4)	C12—C7—C8—C9	0.6 (10)
C7—Sn1—C13—C14	−158.4 (4)	Sn1—C7—C12—C11	179.1 (5)
C7—Sn1—C13—C18	19.7 (5)	C8—C7—C12—C11	−0.5 (9)
C19—Sn1—C13—C14	17.5 (5)	C7—C8—C9—C10	−0.7 (12)
C19—Sn1—C13—C18	−164.4 (4)	C8—C9—C10—C11	0.5 (12)
O2^i^—Sn1—C13—C14	−68.9 (5)	C9—C10—C11—C12	−0.3 (12)
O2^i^—Sn1—C13—C18	109.3 (4)	C10—C11—C12—C7	0.3 (11)
O1—Sn1—C19—C20	132.0 (5)	Sn1—C13—C14—C15	177.6 (5)
O1—Sn1—C19—C24	−49.1 (5)	C18—C13—C14—C15	−0.6 (9)
C7—Sn1—C19—C20	38.2 (6)	Sn1—C13—C18—C17	−177.7 (5)
C7—Sn1—C19—C24	−142.8 (5)	C14—C13—C18—C17	0.5 (8)
C13—Sn1—C19—C20	−137.9 (5)	C13—C14—C15—C16	−0.2 (10)
C13—Sn1—C19—C24	41.0 (5)	C14—C15—C16—C17	1.1 (10)
O2^i^—Sn1—C19—C20	−50.8 (5)	C15—C16—C17—C18	−1.3 (10)
O2^i^—Sn1—C19—C24	128.2 (5)	C16—C17—C18—C13	0.5 (9)
C7—Sn1—O2^i^—P1^i^	117.5 (5)	Sn1—C19—C20—C21	177.3 (5)
C13—Sn1—O2^i^—P1^i^	−6.6 (4)	C24—C19—C20—C21	−1.7 (10)
C19—Sn1—O2^i^—P1^i^	−123.2 (5)	Sn1—C19—C24—C23	−177.4 (5)
O2—P1—O1—Sn1	63.4 (4)	C20—C19—C24—C23	1.6 (9)
C1—P1—O1—Sn1	−172.4 (4)	C19—C20—C21—C22	1.0 (10)
O1—P1—O2—Sn1^ii^	−174.3 (4)	C20—C21—C22—C23	−0.2 (11)
C1—P1—O2—Sn1^ii^	62.6 (5)	C21—C22—C23—C24	0.1 (11)
O1—P1—C1—C2	−48.3 (6)	C22—C23—C24—C19	−0.8 (10)

Symmetry codes: (i) −*x*−1/2, *y*−1/2, *z*; (ii) −*x*−1/2, *y*+1/2, *z*.

### Hydrogen-bond geometry (Å, °)

**Table:**

*D*—H···*A*	*D*—H	H···*A*	*D*···*A*	*D*—H···*A*
C9—H9···Cg1^iii^	0.95	2.79	3.656 (9)	151
C18—H18···Cg1^ii^	0.95	2.91	3.714 (6)	143

Symmetry codes: (iii) *x*+1/2, *y*, −*z*+1/2; (ii) −*x*−1/2, *y*+1/2, *z*.
